# Electroacupuncture for trigeminal neuralgia with anxiety disorders: study protocol for a randomized controlled trial

**DOI:** 10.3389/fmed.2026.1895922

**Published:** 2026-08-19

**Authors:** Rongrong Li, Yaxuan Xu, Lingyu Zhang, Ning Luo, Jing Sun, Jianqiao Fang

**Affiliations:** 1 Department of Acupuncture and Moxibustion, The Third Affiliated Hospital of Zhejiang Chinese Medical University, Hangzhou, China; 2 The Third Clinical Medical College of Zhejiang Chinese Medical University, Hangzhou, China

**Keywords:** anxiety, electroacupuncture, protocol, randomized controlled trial, trigeminal neuralgia

## Abstract

**Introduction:**

Trigeminal neuralgia (TN) is extremely vulnerable to inducing anxiety disorders in TN patients owing to the severe intensity of pain and recurrent episodes. The efficacy of the routine management of TN with anxiety disorders is still unsatisfactory. Acupuncture has been widely utilized in the treatment of TN for many years; however, the effectiveness of Electroacupuncture (EA) for TN with anxiety disorders remains uncertain. Therefore, this randomized, controlled, single-blinded trial was designed to investigate the efficacy and safety of EA for TN with anxiety disorders.

**Methods:**

This single-center, prospective, randomized controlled trial will recruit 60 TN patients with anxiety. All patients will be randomly assigned to the EA or sham EA group in a 1:1 ratio via a computerized central randomization system. The total trial duration is 18 weeks, including a 2-week baseline phase, a 4-week treatment phase, and a 12-week follow-up phase. Participants who meet the inclusion criteria will receive active EA or sham EA treatment for 60 min, three times a week, for 4 weeks.

**Planned Outcomes:**

The primary outcome is the change of Beck Anxiety Inventory score and Visual Analog Scale score from baseline, while the BPI-Facial scores will be regarded as the secondary outcomes. All adverse effects will also be assessed during the study period. Outcome measures will be assessed at pre-intervention, post-intervention (week 4), and at the end of follow-up (week 16).

**Trial Registration:**

Chinese Clinical Trial Registry: ChiCTR2300079048. This study was registered on December 25, 2023.

## Introduction

Trigeminal neuralgia (TN) is a facial pain disorder characterized by unilateral, transient, and recurrent episodes, and is mainly confined to one or more branches of the trigeminal nerve distribution (1, 2). More recently, there has been a gradual increase in the incidence of TN, with an annual rate of approximately 12.6/100, 000 person-years (3), and higher in females (4). Due to the deep intensity of pain and recurrent

attacks, patients with TN are more susceptible to anxiety (5), which seriously affects their quality of life, and has even been historically referred to as a ‘suicidal disease' (6, 7). Notably, anxiety can further exacerbate the degree of pain, thereby affecting the efficacy of the treatments (8). However, the psychological consequences of TN, especially anxiety, have received remarkably less attention.

Pharmacotherapy and psychotherapy are the main therapies for anxiety and have a significant contribution to the improvement of negative emotions, i.e., anxiety disorder (7, 9, 10). Oral selective serotonin reuptake inhibitors are the first-line pharmacologic treatment for anxiety disorders due to their robust efficacy, although other medications may also be efficacious (11–13). Nevertheless, various adverse effects (AEs) are associated, such as headaches, addiction and suicidality (14, 15). Currently, various psychological treatments for anxiety disorders, i.e., mindfulness-based intervention, acceptance and commitment therapy, psychodynamic therapy, emotion-focused therapy, and dialectical behavioral therapy have been tried (16), while some controversies about the efficacy of these interventions remain (17). Moreover, psychotherapy was limited by the scarcity of psychotherapists and the enormous socio-economic costs (18). Hence, there is an urgent demand to find a superior, cost-effective, and secure alternative treatment that reduces the intensity of pain in patients with TN, while ameliorating anxieties.

Acupuncture, a common treatment in Traditional Chinese Medicine, has been widely used for many years in the management of TN, particularly in patients who are medically refractory (19, 20). Meta-analysis demonstrated that electroacupuncture (EA) may be more effective at reducing pain intensity than carbamazepine (CBZ) or other common therapies (21, 22). Recently, Prof. Fang conducted a randomized controlled trial of EA combined with CBZ in 120 TN patients, further confirming the effectiveness of EA. Additionally, the greater benefit of EA combined with low-dose CBZ (300 mg/d) was also revealed in this study, providing a tangible alternative therapeutic choice for TN who cannot tolerate high-dose CBZ (4). Both acupuncture and EA are effective in treating anxiety on their own or as adjuncts to pharmacological therapy (23). Meanwhile, acupuncture can effectively alleviate the anxiety disorders associated with the symptoms, reduce the anxiety score, and improve the prognosis of the disease (24–26), while the quality of studies examining extremely variable (27). Notably, there is currently no effective therapy for TN accompanied by anxiety, and the effectiveness of EA in the treatment of TN with anxiety remains uncertain.

To date, high-quality evidence regarding the therapeutic effect of EA for TN accompanied by anxiety disorders is still lacking (28). Hence, this RCT aims to investigate the efficacy and safety of EA for TN accompanied by anxiety disorders.

## Methods

### Study design

**Figure 1:**
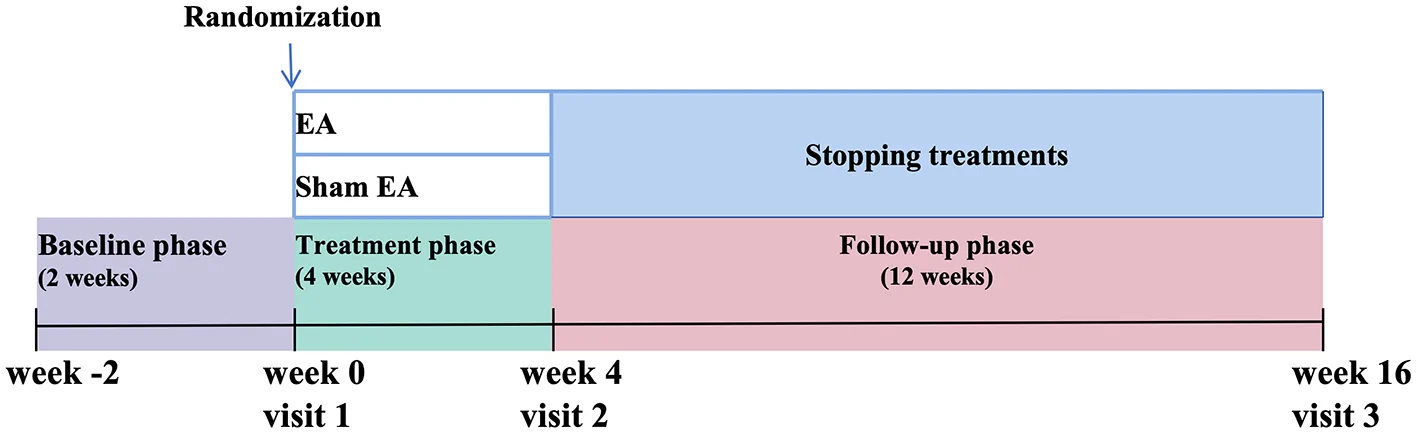
Study design. EA, Electroacupuncture. Rescue medication will be allowed throughout the trial (baseline, treatment phase, follow-up phase), with a maximum daily dosage of 600 mg. The timing and dosage of all rescue medications will be recorded.

**Figure 2:**
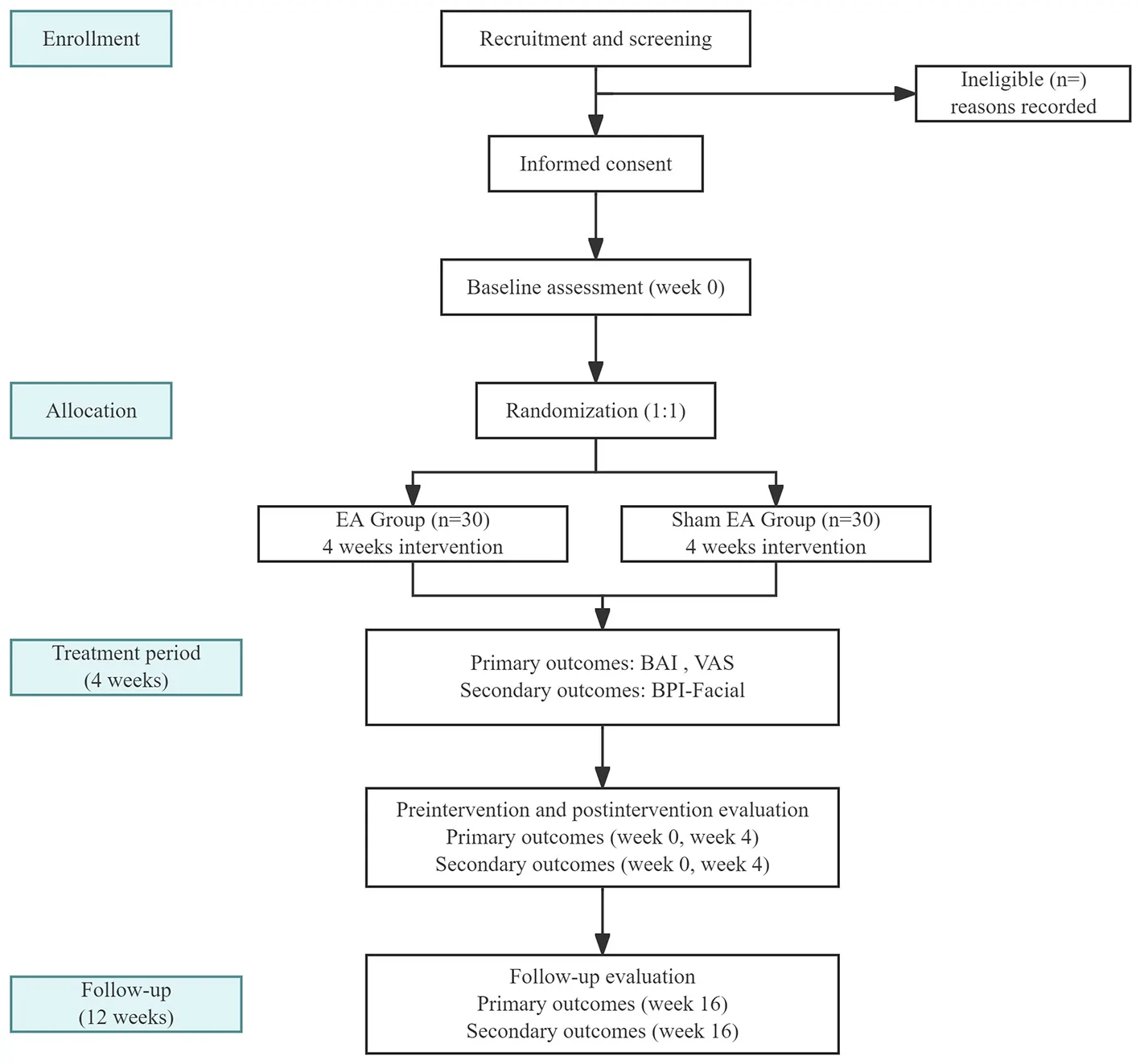
Flowchart of the study process. Bal, Beck Anxiety Inventory; VAS, Visual Analog Scale; BPI Facial, Brief Pain Inventory-Facial Scale.

This single-blind RCT will be conducted at *the Acupuncture Department of the Third Affiliated Hospital of Zhejiang Chinese Medical University*. 60 eligible participants diagnosed with TN comorbid with anxiety will be randomized into two groups: EA and sham EA. The total trial duration is 18 weeks, including a 2-week baseline phase, 4-week treatment phase, and 12-week follow-up phase (Figure 1), and the schedule of enrolment, treatments, and assessments is demonstrated in Table 1. This trial protocol follows the SPIRIT 2025 Statement: Updated Guideline for Protocols of Randomized Trials (29), and the modified CONSORT flow diagram is shown in Figure 2.

**Table 1:** Schedule of enrolment, treatments, and assessments.

Study period	Screening	Baseline	Treatment period	Follow-up period
		Week−2-0	Week 4	Week 16
Eligibility screening	^*^			
Demographic data	^*^			
Case data	^*^			
History of TN	^*^			
History of treatment	^*^			
Inclusion criteria	^*^			
Exclusion criteria	^*^			
Informed consent	^*^			
Treatment condition			^*^	
Outcome assessment				
(1) BAI		^*^	^*^	^*^
(2) VAS		^*^	^*^	^*^
(3) BPI-facial		^*^	^*^	^*^
Safety assessment			^*^	^*^
Recurrence				^*^

^*^Required; BAI, Beck Anxiety Inventory; VAS, Visual Analog Scale; BPI-Facial, Brief Pain Inventory-Facial Scale.

### Ethical considerations and trial registration

This trial will be conducted in accordance with the Declaration of Helsinki, and ethical approval was obtained from the Clinical Trial Ethics Committee of *the Third Affiliated Hospital of Zhejiang Chinese Medical University* (Approval No.: ZSLL-KY-2023-039-01). All patients need to sign informed consent forms before enrolling in this study. Additionally, the trial has been prospectively registered on the *Chinese Clinical Trials Registry*, with registration number ChiCTR2300079048.

### Eligibility criteria

#### Inclusion criteria

(1) diagnosed with TN meet IHCD-3 criteria (30); (2) shooting pain, electric shock pain, or stabbing pain occurring in one or more branches of the trigeminal nerve; (3) Visual Analog Scale (VAS) score ≥4 at baseline, more than three episodes per day, and occurring on at least 4 days per week; (4) male or female participants, age from 18 to 80 years old; (5) Beck Anxiety Inventory (BAI) score ≥16; (6) patients who are conscious, communicate normally, and willing to sign a written informed consent form.

#### Exclusion criteria

Participants will be excluded for the following conditions: (1) patients with secondary TN (e.g. intracranial tumors, multiple sclerosis, etc.); (2) head injury, epilepsy, or other related neurological disorders; (3) cognitive dysfunction, aphasia, or mental disorders, and pain-impaired psychiatric disorders; (4) patients who are pregnant or breastfeeding; (5) not suitable for EA, such as being fitted with a pacemaker; (6) patients with psychogenic pain disorders. Notably, included patients received free treatment to improve compliance.

The specific inclusion/exclusion criteria of eligible TN patients are listed in Table 2.

**Table 2:** Inclusion criteria and exclusion criteria of TN patients.

Inclusion criteria	Exclusion criteria
(1) Diagnosed with TN meet IHCD-3 criteria (2018)	(1) Patients with secondary TN (e.g., intracranial tumors, multiple sclerosis, etc.)
(2) Shooting pain, electric shock pain, or stabbing pain occurring in one or more branches of the trigeminal nerve	(2) Head injury, epilepsy, or other related neurological disorders
(3) Visual analog scale score ≥4 at baseline, more than three episodes per day, and occurring on at least 4 days per week	(3) Cognitive dysfunction, aphasia, or mental disorders, and pain-impaired psychiatric disorders (4) Patients who are pregnant or breastfeeding
(4) Male or female participants, age from 18 to 80 years old	(5) Not suitable for EA, such as being fitted with a pacemaker
(5) Beck Anxiety Inventory score ≥16	(6) Patients with psychogenic pain disorders
(6) Patients who are conscious, communicate normally, and willing to sign a written informed consent form	

TN, Trigeminal neuralgia; IHCD, International Classification of Headache Disorders; EA, Electroacupuncture.

### Sample size

Sample size estimation was performed by the “*Powerandsamplesize.com*” online website (31). This study is the first to explore the therapeutic effect of EA for TN with anxiety. Based on our pilot data with emotion scores between groups [EA group: 3.9 (1.2); control group: 1.2 (0.8)] by using an independent two-sample *t-*test design, a minimum of 25 participants per group will be required (two-sided α = 0.05, power = 0.8). To account for a 20% dropout rate, the final sample size will be set to 30 per group (total *N* = 60). This ensures adequate power even with potential attrition.

### Randomization and blinding

Participants will be screened strictly according to the diagnostic criteria, inclusion and exclusion criteria for TN formulate in this trial. The uniquely eligible participants will be numbered by using IBM SPSS Statistics 25.0 software, the relevant parameters will be entered in the syntax editor, and they will be randomly divided into EA and sham EA groups in a 1:1 ratio. Due to the particularly of the acupuncture treatment, a single-blind method will be applied in this study. Importantly, the patients, outcomes assessor, and statistician will be blinded. To ensure better implementation of the blinding method, each participant will be treated individually to minimize discussion about the acupuncture treatment methods, feelings and the therapeutic effects. We will inform the patients that due to individual difference, each person may experience the stimulation differently. At the end of the 4-week treatment period, participants will be asked to indicate whether they had received real EA or sham EA. The proportions of participants who believed they received real EA will then be compared between the two groups.

### Recruitment strategies

Participants will be recruited from the Third *Affiliated Hospital of Zhejiang Chinese Medical University*, including outpatients and inpatients. Additionally, regular community recruitment will be carried out by measures such as print media and official WeChat accounts.

### Interventions

The location of acupoints are determined in accordance with the People's Republic of China National Standard, “*Interpretation of China national standard Nomenclature and Location of Meridian Points (GB/T 12346-2021)”* (32). Additionally, all acupuncturists are licensed and experienced physicians. Patients will be treated three times per week for 4 weeks, for a total of 12 EA or sham EA sessions.

#### EA group

**Figure 3:**
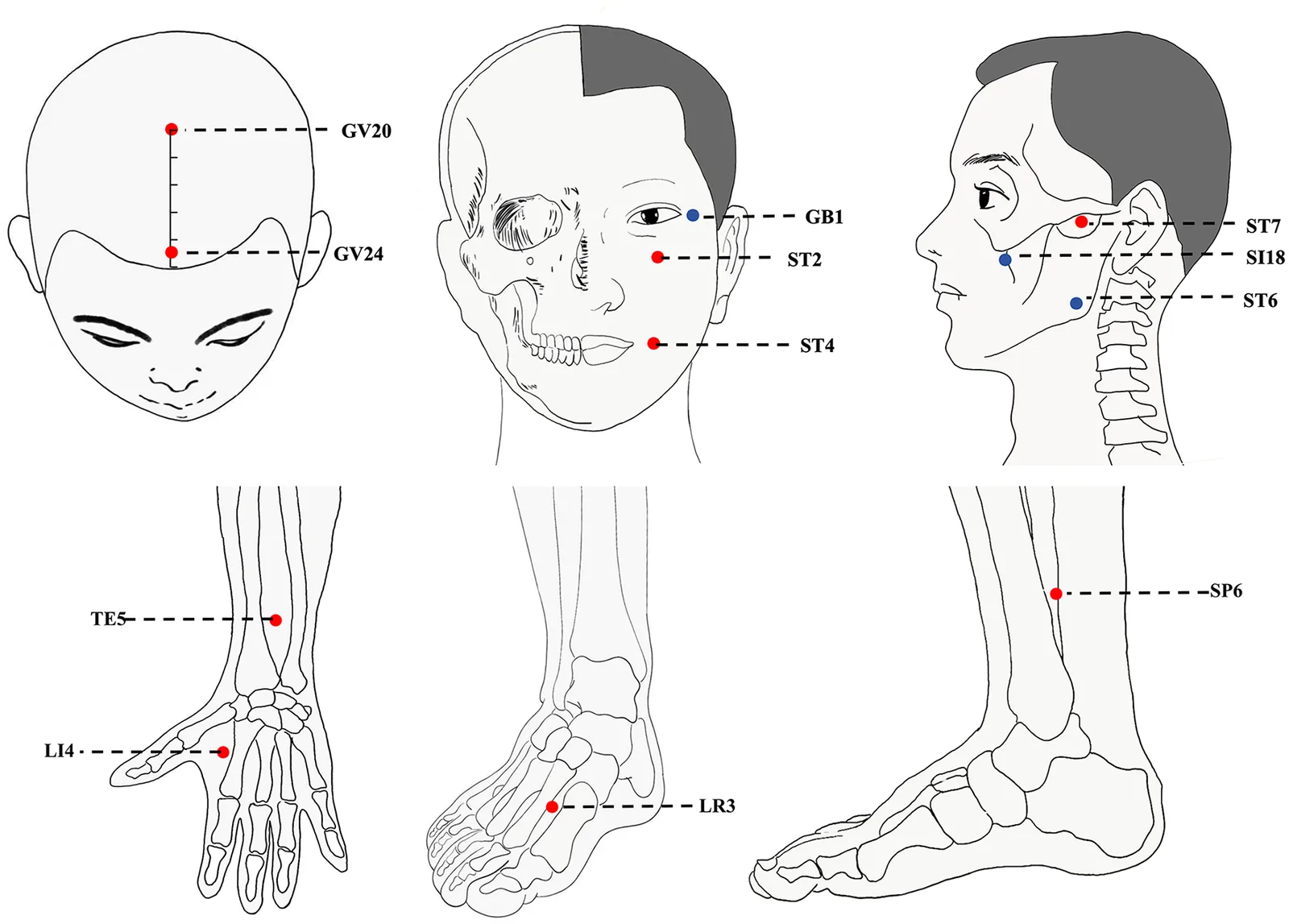
The location of acupoints used in the EA group.

In the EA groups, the mainly local acupoints include Baihui (GV20), Shenting (GV24), Sibai (ST2), Xiaguan (ST7), and Dicang (ST4), needles (size 0.18 mm in diameter, 25 mm in length, Hwato, Co., Ltd., Beijing, China) will be inserted. In addition, Tongziliao (GB1), Quanliao (SI18), and Jiache (ST6) will be selected to match pain in ophthalmic, maxillary, or mandibular branches, respectively. The distal acupoints will be selected by needling (size 0.25 mm in diameter, and 40 mm in length, Hwato, Co., Ltd., Beijing, China) bilateral Hegu (LI4), Waiguan (TE5), Taichong (LR3), and Sanyinjiao (SP6), as shown in Figure 3. Moreover, location of acupoints for treating TN is shown in Table 3. Notably, lesions in the ophthalmic, maxillary, or mandibular branches will be treated with EA treatment, ST7/GB1, ST7/SI18, or ST7/ST6 by using the FANGs acupuncture point nerve stimulator (FANGs-100, Hangzhou Hercules Medical Device Co., Ltd.). The distal acupoints of LI4 and TE5 were also selected. EA frequency alternating waves 2 Hz and 100 Hz will be utilized, with a treatment time of 60 min and a current intensity of 0.5 mA to 1 mA, depending on the individual's needs.

**Table 3:** Acupoints, stimulation, and location.

Category	Acupoints	Stimulation	Location
**Main**	GV20	MA	5 cun directly above the midpoint of the anterior hairline, at the midpoint of the line connecting the apexes of the two auricles
	GV24	MA	0.5 cun directly above the midpoint of the anterior hairline
	ST2	MA	At the infraorbital foramen
	ST4	MA	About 0.4 cun lateral to the corner of the mouth, directly below the pupil of the eye
	ST7	EA	Anterior to the ear, in the depression between the zygomatic arch and the condyloid process of the mandible
Localization of the TN attack			
The 1st trigeminal division	GB1	EA	0.5 cun lateral to the outer canthus on the lateral side of the orbit
The 2nd trigeminal division	SI18	EA	Directly below the outer canthus of the eye, in the depression on the lower border of zygoma
The 3rd trigeminal division	ST6	EA	One finger—breadth (middle finger) anterior and superior to the lower angle of the mandible, where master attaches, at the prominence of the muscle when teeth are clenched
**Distal**	TE5	EA	On the dorsal aspect of the forearm, 2 cun above the transverse crease of the wrist between the ulna and radius
	LI4	EA	On the dorsum of the hand, in the middle of the 2nd metacarpal bone on the radial side
	LR3	MA	On the dorsum of the foot, in the depression proximal to the first metatarsal space
	SP6	MA	3 cun above the medial malleolus, on the posterior border of the medial aspect of tibia

The location will be determined by the People's Republic of China National Standard, *ST7/GB1, ST7/SI18*, or *ST7/ST6* will be chosen for lesions in the 1st trigeminal division (ophthalmic branch), the 2nd trigeminal division (maxillary branch), or the 3^rd^ trigeminal division (mandibular branch) respectively, which will receive EA treatment. GV, Governor Vessel; ST, Stomach; GB, Gallbladder; SI, Small Intestine; TE, Triple Energizer; LI, Large Intestine; LR, Liver; SP, Spleen; MA, Manual Acupuncture; EA, Electroacupuncture.

#### Sham EA group

Participants in the sham EA group will receive needle insertion at non-acupoint sites located 2 cm lateral to the corresponding real acupoints, to a depth of 0.5 mm. Electrode wires that are externally intact but internally disconnected (to prevent current flow) will be used; these wires are carefully inspected and replaced for each participant. The wires are connected to the EA device and attached to non-acupoints in the vicinity of ST7/GB1, ST7/SI18, or ST7/ST6, and LI4/TE5. The device is equipped with external insulation and current leakage protection, and all equipment undergoes pre-use safety checks. To maintain blinding, the device screen and indicator lights will display the same parameters as the active treatment, but no electrical current will be delivered.

The selection of acupoints (local and distal point combination scheme), the 60-min treatment duration per session, and the selection of EA parameters with an alternating frequency of 2/100 Hz were based on our previous research (4, 33).

### Rescue medications

During all three phases of this study, including the 2-week baseline period, 4-week treatment period, and 12-week follow-up period, participants will not receive any treatment other than rescue medication. However, rescue medication will be allowed according to the Declaration of Helsinki for patients experiencing intractable pain. Participants will be allowed to take 200 mg of CBZ after meals, with a maximum dose of 600 mg per day in this study. Rescue medication was administered if the VAS score was ≥5 and persisted for more than 2 h. For each administration, the time and dose were recorded in detail. These recording procedures were predefined.

### Outcome assessment

#### Primary outcomes

This study adopted dual primary endpoints: changes in BAI score and VAS score from baseline to week 4 post-treatment and week 16 follow-up phase, to fully assess the proposed “dual regulatory effect” of EA in TN patients with anxiety.

The BAI is a widely used anxiety scale for psychological assessment of patients and self-assessment in the general population. It was created by the team of American scholar Aaron T. Beck in 1988, with a total of 21 self-report questions (34). Notably, the scale is primarily designed to measure the degree of anxiety and to evaluate the therapeutic effects of anxiety treatment. The BAI adopted the 4-point Likert scale, ranging from 0 to 3 points for each question, based on the following criteria: ‘0′ means not at all; ‘1′ means mildly, with little annoyance; ‘2′ means moderately, feeling uncomfortable but tolerable; ‘3′ means severely, unbearable. The total score is the sum of all questions, ranging from 0 to 63 points, with a higher total score indicating a greater degree of anxiety. Generally, a participant with a total score between 0 to 7 points is considered to have basically no anxiety symptoms, while 8 to 15 points means mild anxiety, 16 to 25 points is moderate anxiety, and above 26 by severe anxiety.

Pain intensity will be evaluated using a 10-cm VAS anchored by ‘0′ (no pain) on the left and ‘10′ (worst pain imaginable) on the right. Participants will be asked to mark the point on the line that best represents their current pain intensity. The distance in centimeters from the ‘0′ end to the mark will be measured and recorded as the pain score. The *International Association for the Study of Pain (IASP)* recommends using the VAS score to assess pain intensity in patients with neuropathic pain, and to validate pain intensity relief with a particular therapy in a clinical trial (Grade A) (35).

#### Secondary Outcome

Secondary outcomes included: (1) Changes in BPI-Facial score from baseline to both the week 4 post-treatment and week 16 follow-up phase. The BPI-Facial is a multidimensional pain scale that captures three interference domains: pain intensity, general interference and facial interference. The total score ranges from 0 to 70, with higher scores indicating a greater impact on quality of life (36). (2) Safety assessment. Adverse events occurring during the study, including acupuncture-related syncope, needle breakage, hematoma, and infection, will be recorded in detail along with the corresponding management strategies. If a serious adverse event occurs, the study intervention will be discontinued immediately. Emergency measures will be taken, and the Ethics Committee of the Third Affiliated Hospital of Zhejiang University of Traditional Chinese Medicine will be notified promptly. All adverse events will be classified and reported in accordance with the CONSORT Harms Reporting Guidelines (37).

### Statistical methods

Statistical analyses will be performed using SPSS version 25.0. Baseline characteristics will be summarized for all randomized participants. The modified ITT population will now serve as the primary analysis group, defined as all randomized participants who received at least one treatment session, regardless of protocol adherence or dropout (in line with CONSORT guidelines). Missing data will be handled via multiple imputation (for continuous outcomes) or worst-case sensitivity analysis (for categorical outcomes). Besides, a per-protocol analysis will be reported as supplementary material to assess robustness. Notably, rescue medication (CBZ) use will be included as a time-varying covariate in primary analyses (e.g., mixed-effects models or ANCOVA) to control for its impact on BAI scores. Additionally, sensitivity analyses will stratify participants by rescue medication status (CBZ users vs. non-users) to evaluate whether treatment effects persist independently of CBZ exposure.

Continuous variables will be expressed as mean (SD) percentile otherwise. For measurement data, normality is first assessed. Normally distributed data will be analyzed using *t-*tests, paired *t-*tests, analysis of variance, or analysis of covariance, while non-normally distributed data will be analyzed using *rank-sum* tests (e.g., *Mann-Whitney U* or *Wilcoxon signed-rank* tests). Categorical variables will be analyzed using *chi-square* or *Fisher's* exact tests.

## Discussion

TN, recognized as one of the most severe neuropathic pain, is characterized by episodic and intense pain. Studies have shown that approximately 45~60% of TN patients exhibit notable anxiety symptoms (38), thereby establishing a vicious cycle of “pain-anxiety-exacerbated pain”. This comorbid state significantly impairs patients' quality of life and decreases the effectiveness of conventional treatments (5, 28).

The following threefold dilemma is currently encountered by clinical treatment. First, pharmacological therapy is limited. CBZ as a first-line treatment for TN is restricted due to some inevitable AEs. Approximately 40%~60% of patients have reported AEs, such as cognitive dysfunction, somnolence, dizziness, gastrointestinal distress, headache, dry mouth, taste disturbances, and mood swings (39). The incidence of AEs increased in up to 55% of patients during dose escalation (40), with some patients discontinuing treatment due to poor tolerability. Second, surgical treatment can cause serious complications. The use of microvascular decompression as the procedure of choice for classic TN is still limited by specific complications and inadequate long-term outcomes, despite its popularity. Studies suggested an incidence of hearing impairment of approximately 1.8%, and cerebrospinal fluid leakage ranging from 1.5% to 4%, following microvascular decompression (41, 42). Poor long-term pain control has also been reported (40). Third, the efficacy of psychotherapy is limited. Cognitive behavioral therapy is the most well-studied and effective psychosocial treatment for depression and anxiety (43), however, its efficacy for treating anxiety specifically related to pain is restricted (44). Furthermore, psychotherapeutic treatment is also under-resourced (43, 45).

EA enhances pain relief and diminishes anxiety scores among patients with TN (4, 46). The mechanism may be related to the following reasons: (1) Through the modulation of opioid peptides, 5-hydroxytryptamine, norepinephrine, glutamate receptors and their transporters, cytokines, and signaling molecules, EA collectively inhibits nociception and emotional responses (47). Among them, opioid peptides play a central role in EA-induced suppression of various types of pain (48–50). (2) The anterior cingulate cortex (ACC) occupies a pivotal position in the interaction between pain and anxiety (51). Evidence shows that EA alleviated pain-related anxiety by inhibiting the PKCzeta/protein kinase Mzeta-GluR1 pathway in the ACC and increasing the n-methyl-d-aspartate receptor pathway in the prefrontal cortex, hippocampus and hypothalamus (52, 53). Further, pain-induced anxiety-like behaviors could also be reduced by EA treatment by activating the rostral ACC-thalamus circuit, suppressing the dopamine D2 receptor pathway in basolateral amygdala (54). (3) The hippocampus is really important for controlling pain and mood (55). Injury and inflammation, for example, can cause changes to the structure and function of hippocampal synapses by activating pain circuits for a prolonged, which can then lead to anxiety-like behaviors (56, 57). Liu et al. showed that EA can encourage the repair and remodeling of hippocampus neurons by increasing the level of factors related to hippocampus' nucleus, balancing iron metabolism and decreasing lipid peroxidation (58). Furthermore, EA reversed synaptic damage by restoring dendritic density and morphology in the hippocampus, producing analgesic and anxiolytic effects (59). In summary, EA achieves simultaneous improvement of pain relief and emotional disorders by acting synergistically on the peripheral, central and limbic systems at multiple targets, particularly by modulating key neuralcircuits.

The EA intervention in this protocol is based on our group's prior clinical studies of EA for TN (4, 33). These studies have demonstrated that EA is superior to CBZ in reducing pain intensity, enhancing facial motor function, and improving quality of life in patients with TN. The protocol also complies with the Evidence-Based Clinical Practice Guidelines for Acupuncture: TN (T/CAAM 018-2015) (60). Local acupoint stimulation acts directly on the trigeminal nerve pathway. It produces analgesia through C-fiber convergence in the nucleus of the solitary tract, stimulates the nerve trunk to promote endorphin release, and improves microcirculation (61, 62). Additionally, EA can modulate synaptic plasticity in the hippocampal CA1 region, thereby alleviating orofacial pain and anxiety (59). The use of alternating 2 Hz and 100 Hz stimulation stimulates the release of multiple opioid peptides, including enkephalins, β-endorphin, and dynorphin, thereby enhancing the analgesic effect (63).

A rescue dose of CBZ up to 600 mg/day was permitted for breakthrough pain management to prevent treatment discontinuation. While EFNS guidelines recommend a therapeutic range from 200 to 1,200 mg/day (64–66), doses exceeding 600 mg/day increase AEs, including drowsiness, dizziness, and postural instability, etc. A study of 354 patients with TN reported a 43.6% AEs incidence at 600–800 mg/day (67), consistent with previous safety reports(64). Therefore, clear criteria was established in this trial for rescue medication administration, and all medication use was documented in detail. Rescue medication doses will be included as covariates in the analysis to control for potential confounding.

Currently, the evidence for the effectiveness of EA in treating TN with anxiety remains insufficient, and the core innovations of this trial are reflected in the following three aspects. Firstly, this study is the first to systematically evaluate the dual regulatory effect of EA on the core symptoms (pain) of TN and its accompanying symptoms (anxiety). Secondly, precise treatment plans will be provided and the combination of acupoints optimized. In accordance with the branch theory of trigeminal neuropathy, this study will innovatively design a series of “local and distal” acupoint combinations. The program will focus on the localization of the lesion, and distant endpoints will also be integrated with systemic regulatory effects to achieve a comprehensive intervention for TN symptoms. Finally, the treatment parameters will be optimized to improve clinical efficacy. 2/100Hz alternating frequency mode will be used in this study, which has been proved to be effective in promoting the release of endogenous analgesic substances, such as β-endorphin (68, 69), possessing a better analgesic effect, nonetheless its therapeutic effects for pain with anxiety have not been clarified. Hence, this study aims to provide evidence of a high standard and innovative clinical treatment strategy for the treatment of TN comorbid anxiety by employing the above-described innovative design.

## Limitations

This study has several unavoidable limitations. First, due to the inherent specificity of the acupuncture procedure, blinding the acupuncture operator will not be feasible in our current research design. To mitigate the influence of subjective factors on study outcomes, a third-party evaluator, who are blinded to subgroup allocations, will be engaged to assess results and conduct statistical analysis, thereby enhancing objectivity and impartiality. Nevertheless, this remains a limitation in the experimental design, warranting optimization in future studies. Additionally, a fixed EA protocol will be employed instead of individualized treatment plans based on practitioner experience, potentially introducing performance bias. However, this standardized approach ensured stringent quality control throughout the trial.

## Conclusions

This protocol describes a rigorously designed, randomized, controlled, assessor-blinded trial designed to investigate the efficacy and safety of EA for TN with anxiety disorders. The results of this study will determine whether EA can alleviate the pain intensity while reducing anxiety symptoms for TN. These findings will be pertinent in determining complementary treatment options for TN with anxiety disorders in clinical practice.
